# Threshold Voltage Adjustment by Varying Ge Content in SiGe p-Channel for Single Metal Shared Gate Complementary FET (CFET)

**DOI:** 10.3390/nano12203712

**Published:** 2022-10-21

**Authors:** Chong-Jhe Sun, Chen-Han Wu, Yi-Ju Yao, Shan-Wen Lin, Siao-Cheng Yan, Yi-Wen Lin, Yung-Chun Wu

**Affiliations:** Department of Engineering and System Science, National Tsing Hua University, Hsinchu 30013, Taiwan

**Keywords:** complementary FET (CFET), threshold voltage adjustment, SiGe, CMOS inverter, technology computer-aided design (TCAD)

## Abstract

We have demonstrated the method of threshold voltage (V_T_) adjustment by controlling Ge content in the SiGe p-channel of N1 complementary field-effect transistor (CFET) for conquering the work function metal (WFM) filling issue on highly scaled MOSFET. Single WFM shared gate N1 CFET was used to study and emphasize the V_T_ tunability of the proposed Ge content method. The result reveals that the Ge mole fraction influences V_TP_ of 5 mV/Ge%, and a close result can also be obtained from the energy band configuration of Si_1-x_Ge_x_. Additionally, the single WFM shared gate N1 CFET inverter with V_T_ adjusted by the Ge content method presents a well-designed voltage transfer curve, and its inverter transient response is also presented. Furthermore, the designed CFET inverter is used to construct a well-behaved 6T-SRAM with a large SNM of ~120 mV at V_DD_ of 0.5 V.

## 1. Introduction

The semiconductor logic device architectures continue to progress, and innovation is driving Moore’s law scaling. Given the transition from planar metal oxide semiconductor field-effect transistor (MOSFET) to three-dimensional FinFET and following stacked nanosheet gate-all-around FET (GAAFETs), the complementary FET (CFET) has been recently proposed as a candidate architecture for the beyond technology node [1,2,3,4]. However, due to shrinking gate length (L_G_), insufficient space for filling multiple work function metal (WFM), which is used for obtaining the desirable device’s threshold voltage (V_T_), has become a challenging problem. Since the gate stack also uses space on the sidewalls under the replacement metal gate (RMG) process [5,6,7], the issue could worsen on stacked nanosheet GAAFETs and become more severe with CFETs. That is because the vertical spacing between the channels must also simultaneously be considered for stacked architectures [8]. As for CFETs, the dual WFM gates should be achieved in the same area but in different layers, which increases the issue’s complexity to a greater extent [1,9].

To maintain the flexibility of multi-V_T_ for balancing low power consumption and high performance, volume-less (also called zero-thickness) methods for VT adjustment are needed. Some research focused on finding methods to reduce the thickness of gate stacks while not losing the V_T_ stability [6,7]. Others proposed an alternative way of using a dipole layer to adjust V_T_ due to its role as an intrinsic fixed charge in gate stacks [10,11]. However, the incorporation of the dipole layer may cause an increase of interface trap density [12,13], which would deteriorate the reliability of the device. Furthermore, the dipole layer may also bring the degradation of mobility [10,13]. This paper proposes another alternative method for adjusting V_T_ by controlling the Ge content in the Si_1-x_Ge_x_ channel. Si_1-x_Ge_x_ is used in strain engineering for hole mobility improvement due to its compatibility in the Si CMOS process [14]. On the other hand, Si_1-x_Ge_x_ was proposed to lower PMOS V_T_ by band engineering [15]. In addition, a FinFET CMOS technology of Si NMOS-SiGe PMOS with common WFM was demonstrated [16]. Additionally, decreasing V_T_ was found in Si_1-x_Ge_x_ PMOS with increasing Ge content [17]. These studies showed that the Si_1-x_Ge_x_ possesses the potential to influence V_T_. Therefore, in this work, we discuss the methodology of V_T_ adjustment by varying Ge content in the SiGe channel and demonstrate the V_T_ adjustment method on the single WFM shared gate CFET as N1 technology using Sentaurus technology computer-aided design (TCAD). The process on the SiGe channel would have no occupation on the spacing for high-k metal gates and gate contact filling. On the other hand, the SiGe channel with Ge content of less than 50% has already been used in today’s semiconductor technology; hence, the proposed method is compatible with the manufacturing technology. In addition, we present the inverter characteristics of the designed N1 CFET and further use it to construct a 6T-SRAM.

## 2. Device Structure and Simulation Methodology

**Figure 1:**
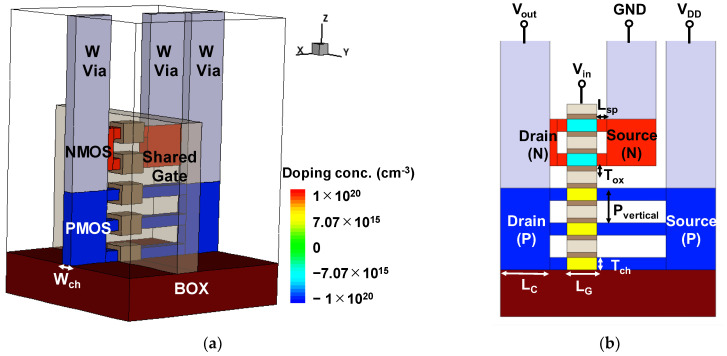
(**a**) The 3-D device structure of CFET and (**b**) the CFET’s cross-sectional view on the *y*-axis cutting plane sitting in the middle of the channel.

Figure 1a displays a bird’s eye view of the N1 CFET architecture in this study. The Synopsys TCAD simulator was employed for the 3-D simulations [18]. The simulation parameters, including gate length (L_G_ = 12 nm), channel thickness (W_ch_ = 6 nm), gate oxide thickness (T_ox_ = 2 nm, HfO_2_), channel vertical pitch (P_vertical_ = 14 nm), spacer length (L_sp_ = 4 nm), and source/drain contact length (L_C_ = 20 nm), are based on the prediction of the 1 nm node logic device in the international roadmap for devices and system (IRDS) 2020 [19].

In the meantime, the architecture designs are also referred from the experimental CFET structure proposed by Intel [1], which stacked the two-channel NMOS on top of the three-channel PMOS on a Si-on-insulator (SOI) substrate. The PMOS is designed to have a longer contact gate pitch (CGP) than the NMOS to separate the source contacts of NMOS and PMOS as the electrodes of GND and V_DD_ for the CFET inverter, respectively. As shown in Figure 1b, the contact of V_in_ is shared by the NMOS gate and the PMOS gate. In addition, V_out_ is shared by the NMOS drain and PMOS drain. GND and V_DD_ are used for the sources of NMOS and PMOS, respectively. Tungsten is used as the contact metal for all the electrodes. Afterward, SiO_2_ is used as fill for oxide passivation, side-wall spacer, and filler of nanosheets inter-spacing. The structural simulation parameters of the N1 CFET simulation are shown in Table 1.

To increase the accuracy of the simulation in this study, the I_D_-V_G_ transfer characteristics of the CFET with L_G_ of 75 nm were calibrated to the experimental result from [1]. The following physical models were considered and coupled in the TCAD simulation:

1. The drift-diffusion model was included with the coupled Possion’s and continuity equations to determine the electrostatic potential and carrier transport.
2. The density gradient model was included to correct the quantum confinement effect in the drift-diffusion model due to the highly scaled dimension [20].
3. The doping-concentration-dependent Shockley–Read–Hall (SRH) recombination model was included for the generation–recombination mechanism.
4. The Slotboom bandgap narrowing model was included for doping-concentration-dependent bandgap correction [21].
5. The doping-dependent, transverse field dependence, and high-field saturation mobility models were included to consider impurity scattering, interfacial surface roughness scattering, and coulomb scattering degradations.
6. A ballistic mobility model was considered for quasi-ballistic transport.

**Figure 2:**
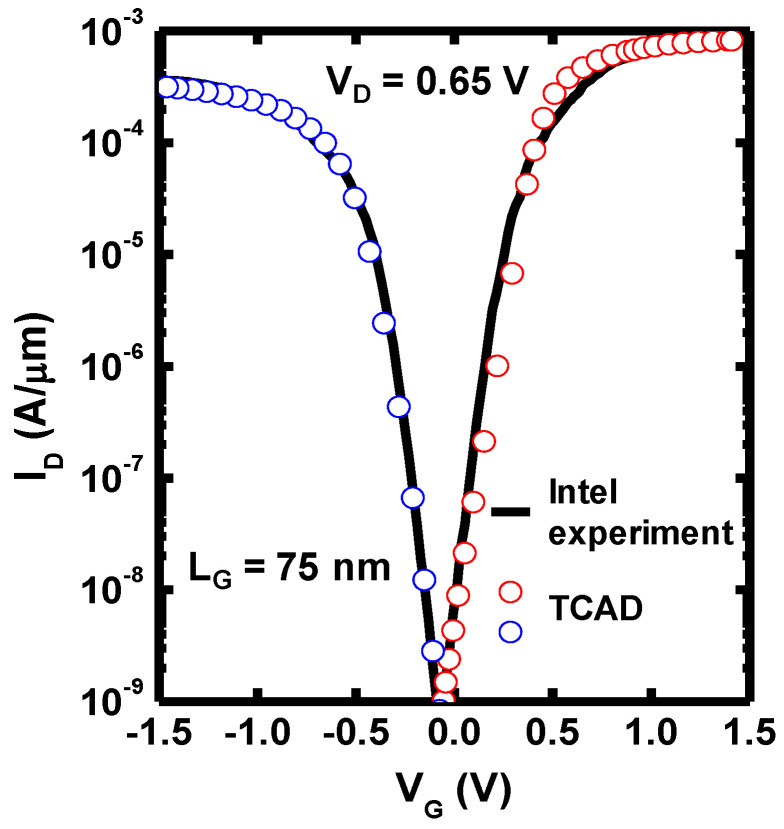
Calibrated I_D_-V_G_ transfer characteristics of CFET between Intel experimental data [1] and TCAD simulation.

The calibration result is shown in Figure 2. The simulation of the following inverter transient response and the 6T-SRAM were achieved using “mixed-mode” in SDEVICE of Sentaurus TCAD.

## 3. Results and Discussion

**Figure 3:**
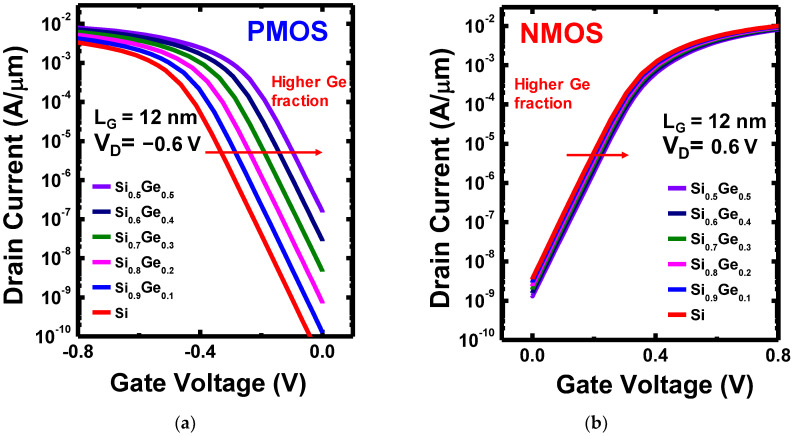
(**a**,**b**) are I_D_-V_G_ transfer curves of the PMOS and NMOS, respectively, in CFET structure, with varying Ge mole fraction (x) from 0 to 50%.

First, to demonstrate the V_T_ tunability of changing the Ge mole fraction (x) in the Si_1-x_Ge_x_ channel for N1 CFET, we analyzed the electrical characteristics of PMOS and NMOS on the Si_1-x_Ge_x_ composition in the CFET structure. Figure 3a,b show the I_D_-V_G_ transfer curves of PMOS and NMOS, respectively, in CFET structure with V_D_ = ±0.6 V. For the individual electrical characteristics of NMOS and PMOS, only the targeted MOS’s corresponding gate, source, and drain were contacted, and the remaining contact was floating. For example, while extracting the electrical characteristics of the NMOS, the V_in_ (gate of the NMOS), GND (source of the NMOS), and V_out_ (drain of the NMOS) were contacted. In addition, V_DD_ was floating. The mole-fraction-dependent material, Si_1-x_Ge_x_, is set as the channel material for both PMOS and NMOS; that is, the channel is Si if x = 0 and Ge if x = 1. As the Ge mole fraction varies from 0 to 0.5, the I_D_-V_G_ curves of both PMOS and NMOS shift to the right. However, by comparison, PMOS shows a more noticeable shift on V_T_. As for NMOS, the shift is relatively negligible. Due to the excellent gate control ability benefitting from the GAA structure, as the x ranged from 0 to 0.5, the subthreshold swing (SS) and drain-induced barrier lowering (DIBL) are nearly unchanged for both NMOS and PMOS. The SS and DIBL are not shown in the figure.

**Figure 4:**
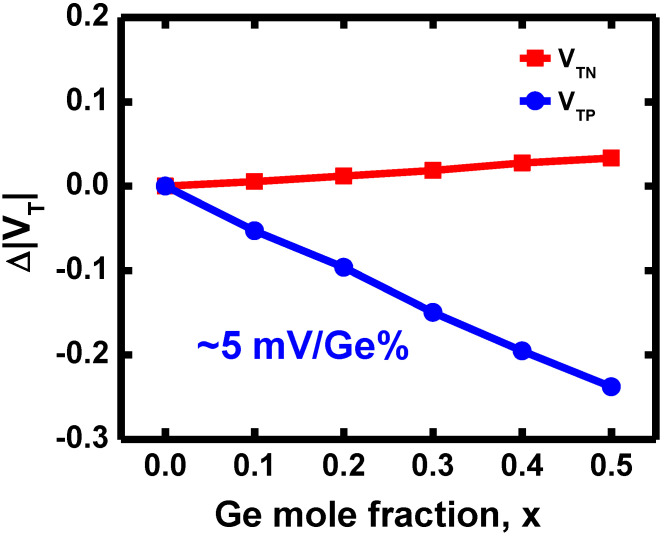
The V_T_ shift of NMOS and PMOS with varying Ge mole fraction, x, where the V_T_ shift is quantified relative to the VT of x = 0.

**Figure 5:**
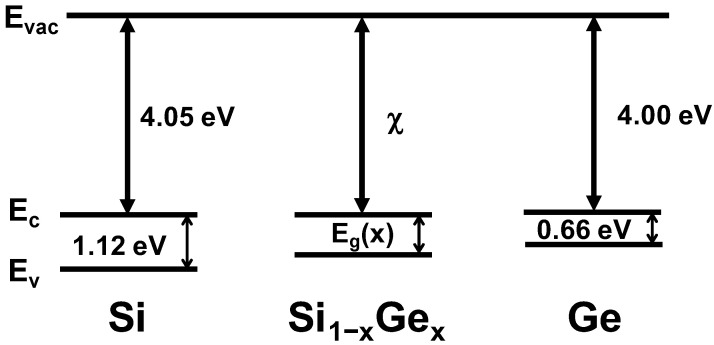
Energy band diagram of Si, SiGe, and Ge.

The V_T_ shift quantified relative to the V_T_ of x = 0 is shown in Figure 4. V_T_ was extracted by the conductance method. V_TN_ has only a 33 mV difference as x increases from 0 to 0.5, whereas the absolute value of V_TP_ decreases linearly with a slope of approximately 5 mV/Ge%. This is because while the electron affinity is 4.05 eV for Si and 4.00 eV for Ge, which are very close, the energy band gaps (E_g_) differ, 1.12 eV for Si and 0.66 eV for Ge [17], as shown by the energy band diagrams of Si, SiGe, and Ge in Figure 5. That gap results in the valence band energy (E_v_) being pulled toward the vacuum level as the Ge incorporates into the Si channel, whereas the conduction band energy (E_c_) remains at nearly the same level.

The E_g_ of Si_1-x_Ge_x_ can be expressed as follows [22]:

E_g_ = 1.12 − 0.41x + 0.008x^2^, x < 0.85, 300 K (1)

Since the E_g_ narrowing of Si_1-x_Ge_x_ is mainly attributed to E_v_ offset, the V_TP_ is more sensitive to the Ge content than V_TN_. In addition, as can be seen from Equation (1), if we neglect the contribution of E_c_ changing and the trivial quadratic term, the E_g_ would have a rate of change of approximately 4.1 meV with respect to the Ge x, which is also very close to the simulation result of the V_TP_ shift.

**Figure 6:**
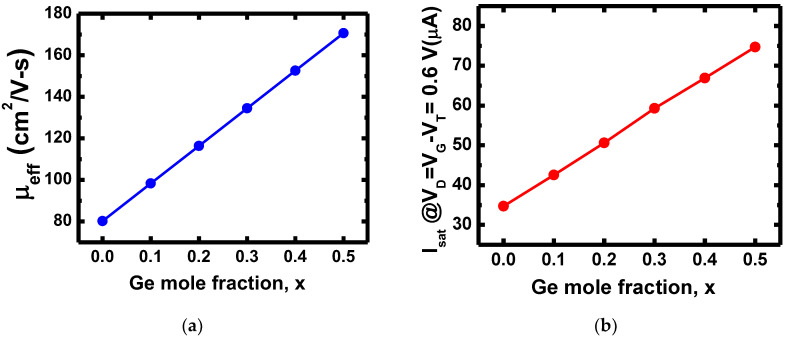
(**a**) extracted effective hole mobility of PMOS with varying Ge mole fractions. (**b**) I_sat_ of PMOS with varying Ge mole fractions. I_sat_ was extracted at V_D_ = V_G_ − V_T_ = 0.6 V.

On the other hand, the adjustment of the threshold voltage by varying Ge content in CFET might result in a change in charge carrier mobility. As shown in Figure 6, we analyzed the effective hole mobility and saturation current (I_sat_) of PMOS. We focus only on PMOS and hole mobility since V_TN_ is not sensitive to varying Ge mole fractions. The I_sat_ was extracted at V_D_ = V_G_-V_T_ = 0.6 V. By increasing the Ge mole fraction in Si_1-x_Ge_x_ p-channel, the effective hole mobility and I_sat_ are both enhanced linearly. The effective hole mobility increases by 112%, and I_sat_ increases by 115% as the Ge mole fraction increases from 0 to 0.5. For Ge mole fraction higher than 0.5, the hole mobility and I_sat_ would be much higher. As a result, it might not be suitable to adjust V_T_ with Ge mole fraction higher than 0.5.

**Figure 7:**
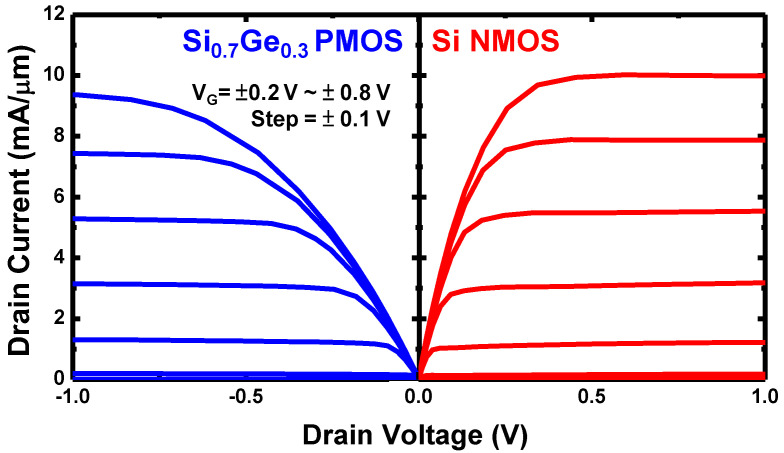
I_D_-V_D_ output characteristic of Si_0_._7_Ge_0_._3_ PMOS and Si NMOS in CFET structure with L_G_ = 12 nm, showing good symmetric output current.

As the V_TP_ is much more adjustable by varying the Ge content, it is justifiable to use Si as the channel material of NMOS and adjust only the V_T_ by changing x in the Si_1-x_Ge_x_ p-channel for designing the N1 CFET inverter. By performing a single WFM (for adjusting V_TN_) and Ge content method (for adjusting V_TP_) together on CFETs, we could relieve the lack of spacing for dual WFM. Therefore, we then tuned the work function of the shared gate of the N1 CFET to let the Si NMOS possess an expected V_TN_. In this case, we set the work function to 4.49 eV to let V_TN_ = 0.25 V. Subsequently, we varied the x of the Si_1-x_Ge_x_ PMOS to match V_TP_ and V_TN_. Notice that the I_D_-V_G_ curves in Figure 3 are with the N1 CFET, whose work function of the shared gate was set. As can be seen from Figure 3, the PMOS with Si_0_._7_Ge_0_._3_ has a V_TP_ of −0.25 V, which matches the Si NMOS. Figure 7 shows the I_D_-V_D_ output characteristics of the Si_0_._7_Ge_0_._3_ PMOS and the Si NMOS in the N1 CFET structure with V_G_ ranging from ±0.2 to ±0.8 V at a step of ±0.1 V. Their output currents are comparable, though the PMOS has a longer CGP which may lead to a more significant total resistance. That implies the design of more stacks of PMOS than NMOS can overcome the degradation.

**Figure 8:**
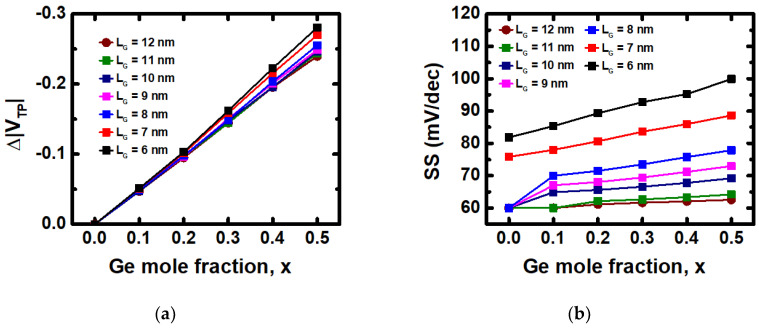
(**a**) V_TP_ and (**b**) SS of Si_1-x_Ge_x_ PMOS with Ge mole fraction from 0 to 0.5, and L_G_ from 12 nm to 6 nm.

Figure 8a,b show the analysis of the electrical characteristics including V_TP_ and subthreshold swing (SS) of Si_1-x_Ge_x_ PMOS with different L_G_ from 12 nm to 6 nm. As L_G_ values shrink down to below 8 nm, the assumption of the 5 mV/Ge% V_TP_ relation would become unsuitable. The assumption shows a deviation of less than 4%, with L_G_ ranging from 12 nm to 8 nm. However, the deviation becomes larger than 10% with L_G_ values of 7 nm and below. In Figure 7b, SS would increase with a higher Ge mole fraction, but the increase is ignorable with L_G_ of 12 nm and 11 nm. As the L_G_ becomes smaller, the increase of SS with respect to Ge mole fraction would then get more obvious, but still acceptable when L_G_ is larger than 8 nm. However, at L_G_ smaller than 8 nm, SS becomes no longer suitable. In conclusion, the proposed Ge content method for adjusting V_T_ can be applied on CFET device with L_G_ scaled down to 8 nm.

**Figure 9:**
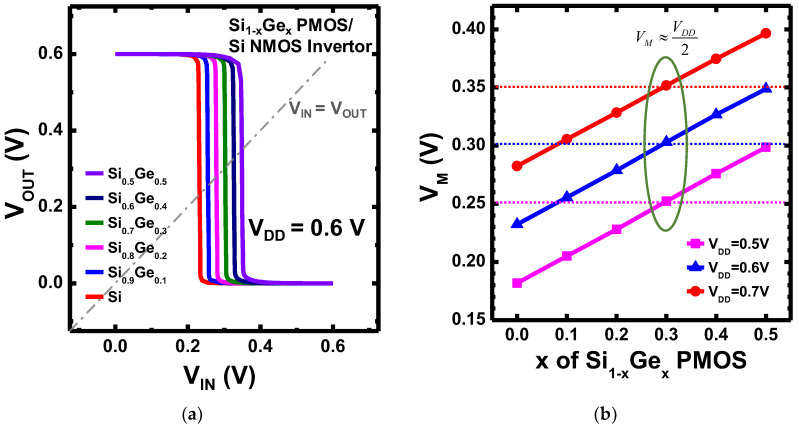
(**a**) The voltage transfer curves (VTC) of CFET inverter with Si_1-x_Ge_x_ PMOS and Si NFET. (**b**) The inverters’ switching thresholds (V_M_) versus x in Si_1-x_Ge_x_ PMOS with V_DD_ = 0.5, 0.6, and 0.7 V, and the dotted lines represent where the V_M_ equals V_DD_/2.

**Figure 10:**
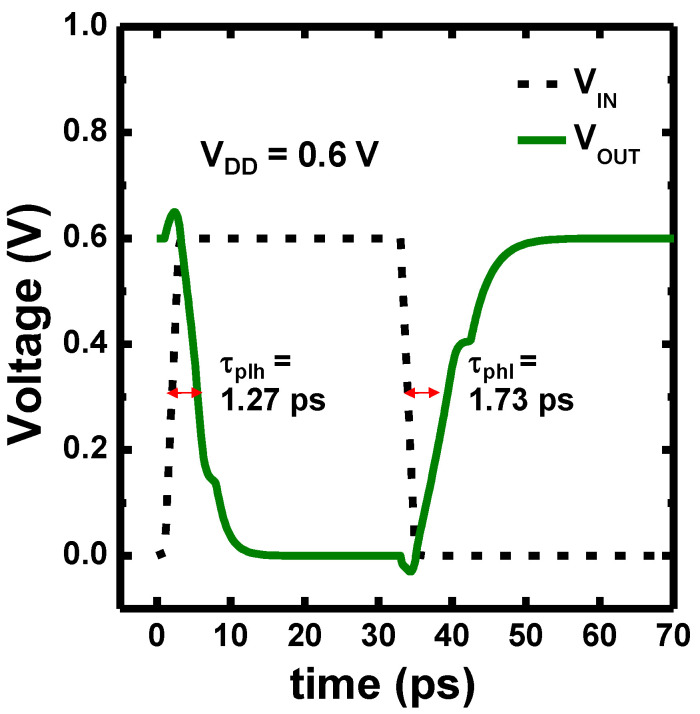
Transient response of the designed N1 CFET inverter with Si_0_._7_Ge_0_._3_ PMOS and Si NMOS under a ~14 GHz operation.

The N1 CFET inverter constructed with Si_1-x_Ge_x_ PMOS and Si NMOS was also analyzed. Figure 9a shows the voltage transfer curves of the N1 CFET inverter with x of the Si_1-x_Ge_x_ PMOS varying from 0 to 0.5 and the V_DD_ of 0.6 V. The switching thresholds (V_M_) of the VTCs were extracted as the voltage where V_IN_ = V_OUT_, as shown in Figure 9b. The V_M_ of the N1 CFET inverter at V_DD_ of 0.5, 0.6, and 0.7 V increase monotonically with increasing x of the Si_1-x_Ge_x_ PMOS. The dotted lines represent where the V_M_ equals V_DD_/2; in this case, the N1 CFET inverter with x = 0.3 has the V_M_ nearly V_DD_/2 for all three V_DD_ due to the V_T_ and current matching. Figure 10 presents the transient response of the designed N1 CFET inverter with Si_0_._7_Ge_0_._3_ PMOS and Si NMOS under a ~14 GHz operation. The transient response was performed with fan-out of 3 (FO3) and without load capacitance. The designed N1 CFET inverter exhibits propagation delay times from low to high (τ_plh_) and from high to low (τ_phl_) of ~1.27 ps and ~17.3 ps, respectively. The propagation delay times were extracted at 0.5 V_DD_.

**Figure 11:**
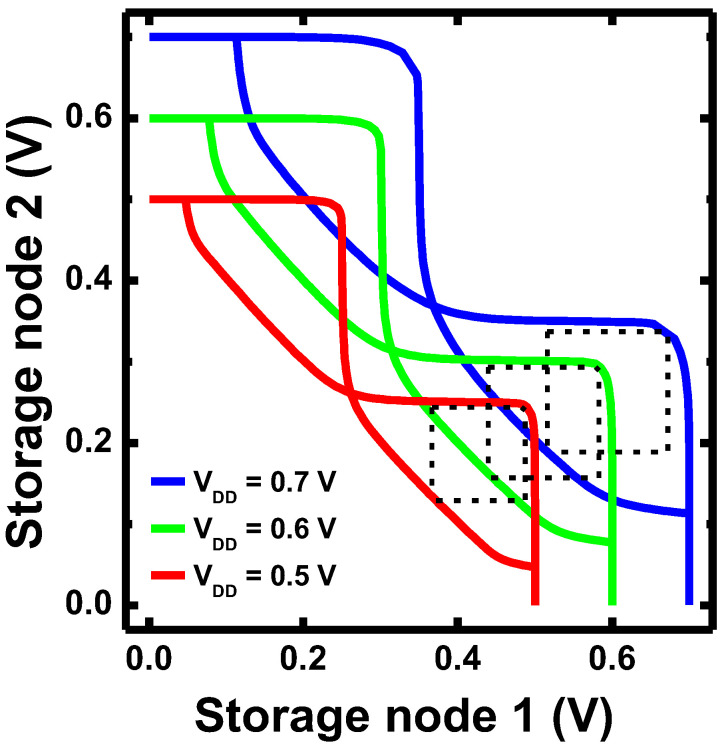
The butterfly curves of the 6T-SRAM cell, which was constructed with two designed N1 CFET inverters and two NMOS access transistors. The SNM of ~120 mV as V_DD_ down to 0.5 V is obtained.

Moreover, the designed N1 CFET inverter with Si_0_._7_Ge_0_._3_ PMOS and Si NMOS was used to build a 6T-SRAM cell. The 6T-SRAM cell was constructed with two N1 CFET inverters and two NMOS access transistors. The butterfly curves of the 6T-SRAM built with N1 CFET inverters at V_DD_ of 0.5, 0.6, and 0.7 V are shown in Figure 11. Excellent stability is present with a large static noise margin (SNM) of ~120 mV as V_DD_ down to 0.5 V, and the SNM values are ~140 and ~155 mV at V_DD_ of 0.6 and 0.7 V, respectively.

## 4. Conclusions

In this study, we proposed the V_T_ adjustment method by controlling Ge content in the SiGe channel and demonstrated it on N1 CFET by TCAD simulation. The PMOS shows a high sensitivity on the Ge mole fraction since the incorporation of Ge pulls E_v_ towards the vacuum level but has little effect on E_c_. The simulation result shows the V_TP_ has a change rate of approximately 5 mV/Ge%, which is close to that derived from the E_g_ relation. The N1 CFET designed by the Ge content method presents a good VTC and nearly V_DD_/2 V_M_. Well-performing inverter transient response is also presented. In addition, the 6T-SRAM shows a large SNM of ~120 mV as V_DD_ down to 0.5 V. With the help of the proposed Ge content method, the V_T_ tuning flexibility can be significantly improved for the highly scaled device.

## Figures and Tables

**Table 1:** Simulation parameters of 1 nm node CFET devices.

Fixed Parameter	Quantity	Value
*W_ch_*	Channel width	6 nm
*T_ch_*	Channel thickness	5 nm
*T_ox_*	Gate oxide thickness (HfO_2_)	2 nm
*P_vertical_*	Channel vertical pitch	14 nm
*L_sp_*	Spacer length	4 nm
*L_C_*	S/D contact length	20 nm
*N_S/D_*	S/D Doping concentration	1 × 10^20^ cm^−3^
*N_ch_*	Channel Doping concentration	1 × 10^16^ cm^−3^
**Variable Parameter**	**Quantity**	**Value**
*x*	Ge mole fraction of Si_1-x_Ge_x_ channel	0–0.5
*L_G_*	Gate length	6-12 nm

## Data Availability

The data presented in this study are available on request from the corresponding author.
